# Reduced naïve CD8^+^ T‐cell priming efficacy in elderly adults

**DOI:** 10.1111/acel.12384

**Published:** 2015-10-15

**Authors:** Olivia Briceño, Anna Lissina, Kerstin Wanke, Georgia Afonso, Amrei von Braun, Kristanto Ragon, Tiphaine Miquel, Emma Gostick, Laura Papagno, Karin Stiasny, David A. Price, Roberto Mallone, Delphine Sauce, Urs Karrer, Victor Appay

**Affiliations:** ^1^ Centre d'Immunologie et des Maladies Infectieuses (CIMI‐Paris)Sorbonne Universités, UPMC Univ Paris 06, DHU FASTCR7F‐75013ParisFrance; ^2^ CIMI‐ParisINSERM, U1135F‐75013ParisFrance; ^3^ Division of Infectious DiseasesUniversity Hospital of ZurichZurichSwitzerland; ^4^ INSERM, U1016Institut CochinParisFrance; ^5^ CNRS, UMR8104ParisFrance; ^6^ Faculté de MédecineUniversité Paris Descartes, Sorbonne Paris CitéParisFrance; ^7^ Institute of Infection and ImmunityCardiff University School of MedicineCardiffWalesUK; ^8^ Department of VirologyMedical University of ViennaViennaAustria; ^9^ Service de DiabétologieAssistance Publique‐Hôpitaux de ParisHôpital CochinParisFrance

**Keywords:** aging, naïve CD8^+^ T‐cells, priming

## Abstract

Aging is associated with impaired vaccine efficacy and increased susceptibility to infectious and malignant diseases. CD8^+^ T‐cells are key players in the immune response against pathogens and tumors. In aged mice, the dwindling naïve CD8^+^ T‐cell compartment is thought to compromise the induction of *de novo* immune responses, but no experimental evidence is yet available in humans. Here, we used an original *in vitro* assay based on an accelerated dendritic cell coculture system in unfractioned peripheral blood mononuclear cells to examine CD8^+^ T‐cell priming efficacy in human volunteers. Using this approach, we report that old individuals consistently mount quantitatively and qualitatively impaired *de novo* CD8^+^ T‐cell responses specific for a model antigen. Reduced CD8^+^ T‐cell priming capacity *in vitro* was further associated with poor primary immune responsiveness *in vivo*. This immune deficit likely arises as a consequence of intrinsic cellular defects and a reduction in the size of the naïve CD8^+^ T‐cell pool. Collectively, these findings provide new insights into the cellular immune insufficiencies that accompany human aging.

## Introduction

Elderly people suffer more often and more severely from infectious and malignant diseases than young individuals. Responses to vaccination are also less effective in the elderly. These manifestations are thought to reflect an age‐related functional decline in the immune system (Dorshkind *et al*., 2009). An integrated understanding of the aging immune system is therefore essential for the delivery of optimal healthcare to the elderly population. Adaptive immunity mediated by T‐cells is critically important in the host response to various pathogens and cells undergoing malignant transformation. However, increasing evidence suggests that age‐related changes limit the efficacy of cellular immune responses against emerging strains of influenza virus and tumor cells. Thus, while recall responses to pathogens encountered earlier in life are largely uncompromised, primary T‐cell‐mediated immune responses appear to decline with advanced age (Nikolich‐Zugich, 2014).

Primary T‐cell responses originate from the naïve T‐cell compartment, which provides a clonotypically diverse reservoir of specific precursors that differentiate upon antigen engagement to populate the memory and effector T‐cell compartments (Stemberger *et al*., 2007). In turn, the naïve T‐cell pool is generated by thymic output. However, the thymus atrophies with age, a process termed ‘thymic involution’ (Gruver *et al*., 2007). This progressive decay in thymic function leads to a corresponding loss of naïve T‐cells and increased homeostatic proliferation within the naive T‐cell compartment (Kohler & Thiel, 2009; Sauce *et al*., 2012). Nonetheless, such enhanced proliferation is insufficient to maintain the number (Sauce *et al*., 2012) and clonotypic diversity (Naylor *et al*., 2005) of peripheral naïve T‐cells in elderly adults. Collectively, these observations indicate that the naïve T‐cell pool becomes quantitatively impaired with advanced age.

Recent studies in animal models further suggest qualitative alterations in the naïve T‐cell compartment and impaired induction of *de novo* T‐cell responses with age (Brien *et al*., 2009; Cicin‐Sain *et al*., 2010; Smithey *et al*., 2011; Renkema *et al*., 2014). Indeed, contraction of the murine naïve T‐cell repertoire can impair T‐cell responses to immunodominant epitopes (Yager *et al*., 2008; Valkenburg *et al*., 2012). Intrinsic age‐related defects in naïve T‐cell responsiveness linked to gene expression profiles and cytokine secretion have also been documented in old mice (Mirza *et al*., 2011; Decman *et al*., 2012; Jiang *et al*., 2013). In contrast, experimental evidence for compromised naïve T‐cell responsiveness in old humans is scarce (Goronzy & Weyand, 2013; Appay & Sauce, 2014). This is an important knowledge gap given that differences have been observed in age‐associated naïve T‐cell dynamics between mice and humans (den Braber *et al*., 2012).

One recent study revealed an alteration in naïve CD4^+^ T‐cell activation and differentiation in the elderly, associated with a defect in TCR‐induced extracellular signal‐regulated kinase (ERK) phosphorylation (Li *et al*., 2012). Age‐related changes in humans have also been described for memory CD8^+^ T‐cells, which provide frontline defence against viruses and tumors (Appay *et al*., 2008). In particular, elderly individuals harbor a higher proportion of terminally differentiated (CD28^−^) memory CD8^+^ T‐cells, which exhibit strong effector functions constrained by limited proliferative capabilities in tandem with short telomeres (reviewed in Pawelec *et al*., 1999). More recently, Akbar and colleagues have provided new insights into the associated molecular defects (Henson *et al*., 2014). However, there is limited information regarding the capacity of old humans to mount primary CD8^+^ T‐cell responses.

Evaluating *de novo* antigen‐specific T‐cell responses in humans is difficult beyond the limited setting of vaccination and primary infection. To overcome this difficulty, we developed an *in vitro* assay to assess naïve CD8^+^ T‐cell priming directly from unfractionated peripheral blood mononuclear cells (PBMCs). This approach is based on an accelerated dendritic cell (DC) coculture system, designed for the optimal activation of antigen‐specific T‐cells from PBMCs (Martinuzzi *et al*., 2011). As circulating human DCs are rare, DC precursors within the starting PBMC material were mobilized using FLT3 ligand (FLT3L) (Breton *et al*., 2015), known for its capacity to enhance the induction of T‐cell responses (Guermonprez *et al*., 2013), and matured with a standard cocktail of inflammatory cytokines (TNF, IL‐1β, PGE2, and IL‐7).Furthermore, we focused on the priming of CD8^+^ T‐cells specific for the melanoma antigen Melan‐A/MART‐1, restricted by HLA‐A*0201 (HLA‐A2 from hereon). This specificity is particularly amenable to *in vitro* studies with limited volume blood samples due to naturally high precursor frequencies in the naive pool and the widespread occurrrence of HLA‐A2 in the general population. Equipped with this original and broadly applicable assay, we set out to obtain further insights into the decline of CD8^+^ T‐cell immunity with age.

## Results

### *In vitro* model of antigen‐specific naïve CD8^+^ T‐cell priming

The frequency of circulating antigen‐reactive CD8^+^ T‐cell precursors in humans is typically very low, often in the order of one cell per million within the lineage as a whole (Alanio *et al*., 2010; Legoux *et al*., 2010; Iglesias *et al*., 2013). To circumvent this biological obstacle to the reliable study of antigen‐specific priming *in vitro*, we exploited the observation that individuals expressing the HLA‐A2 allotype harbor exceptionally high frequencies of precursors (i.e., 10–100 naïve cells per million CD8^+^ T‐cells) specific for the heteroclitic Melan‐A/MART‐1 epitope ELAGIGILTV (ELA from hereon) (Dutoit *et al*., 2002; Zippelius *et al*., 2002). This large antigen‐specific pool in the naïve CD8^+^ T‐cell compartment provides a unique means to study the priming of human T‐cells that recognize ELA, used here as a model antigen (Pinto *et al*., 2014; Romero *et al*., 2014). It is also notable that the Melan‐A/MART‐1 epitope is an important melanoma‐associated antigen. Studying Melan‐A/MART‐1‐specific T‐cell priming is therefore directly relevant to the assessment of antitumor immunity in the elderly. Combined with the high frequency of the HLA‐A2 allotype in the Caucasian population (i.e., ~45% prevalence), this approach enabled reproducible *in vitro* priming using a small number of PBMCs (5 × 10^6^ in our assays) from a large number of (HLA‐A2^+^) individuals, in response to stimulation with the cognate ELA epitope encompassed within a longer (i.e., 20‐mer) synthetic peptide.

**Figure 1:**
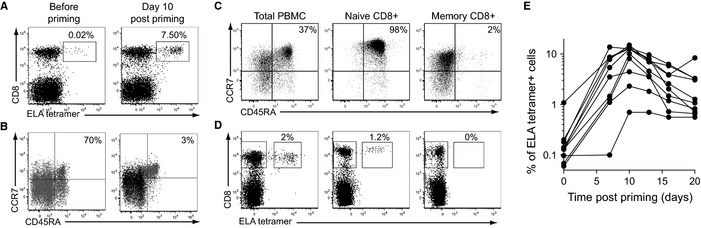
*In vitro* priming of antigen‐specific CD8^+^ T‐cells from naïve precursors. (A) Representative flow cytometry plots showing ELA/HLA‐A2 tetramer staining of donor PBMCs before (day 0) and after (day 10) priming. Percentages of ELA/HLA‐A2 tetramer^+^ cells within the CD8^+^ T‐cell population are indicated. (B) Representative phenotype of ELA/HLA‐A2 tetramer^+^ (black) or total (gray) CD8^+^ T‐cells at day 0 and at day 10 postpriming. Percentages of ELA/HLA‐A2 tetramer^+^ naïve CD8^+^ T‐cells (CD45RA^+^ CCR7^+^) are shown. (C) Representative flow cytometry plots showing the phenotypes of total, naïve, and memory purified CD8^+^ T‐cells used for *in vitro* priming. Percentages of naïve CD8^+^ T‐cells (CD45RA^+^ CCR7^+^) are indicated. (D) Tetramer staining of ELA‐specific CD8^+^ T‐cells at day 10 postpriming is shown for each of the starting populations depicted in (C). Purified naïve and memory CD8^+^ T‐cell populations were supplemented separately with autologous CD8‐depleted PBMCs to initiate priming. Percentages of ELA/HLA‐A2 tetramer^+^ cells within the CD8^+^ T‐cell population are indicated. Data shown are representative of three independent experiments. (E) Expansion kinetics of ELA/HLA‐A2 tetramer^+^ CD8^+^ T‐cells after antigen‐specific priming of PBMCs from 10 different healthy donors.

Upon priming from total PBMCs with a stimulation cocktail incorporating the ELA peptide, FLT3L, TNF‐α, IL‐1β, PGE2, and IL‐7 (Martinuzzi *et al*., 2011), ELA‐specific CD8^+^ T‐cells were quantified by flow cytometry using fluorochrome‐labeled ELA/HLA‐A2 tetramers (Fig. 1A), and their differentiation phenotype was assessed according to CD45RA and CCR7 surface expression (Fig. 1B). After expansion, the majority of ELA‐specific CD8^+^ T‐cells exhibited a memory phenotype (CD45RA^−^ CCR7^+/−^), reflecting the differentiation of ELA‐reactive naïve CD8^+^ T‐cell precursors (CD45RA^+^ CCR7^+^). No expansion of ELA‐specific CD8^+^ T‐cells was observed priming in the absence of peptide (data not shown). Although it is established that ELA‐reactive CD8^+^ T‐cells in healthy donors can be defined as naïve T‐cells (characterized by a CD45RA^+^ CCR7^+^ phenotype, a high TREC content and long telomeres) (Dutoit *et al*., 2002; Zippelius *et al*., 2002), we confirmed that ELA‐specific T‐cell priming in these donors was indeed occurring within the naïve (and not memory) CD8^+^ T‐cell compartment. For this purpose, we mixed purified naïve or memory CD8^+^ T‐cells separately with autologous CD8‐depleted PBMCs to initiate priming (Fig. 1C). Antigen‐specific expansion was only observed with naïve CD8^+^ T‐cells (Fig. 1D), thereby validating the experimental system. Optimization experiments with healthy donor PBMC samples revealed maximal ELA‐specific CD8^+^ T‐cell expansion at 10–11 days postpriming (Fig. 1E). This time course was adopted in all subsequent assays.

### *In vitro* CD8^+^ T‐cell priming as a correlate of *de novo* immune responsiveness

**Figure 2:**
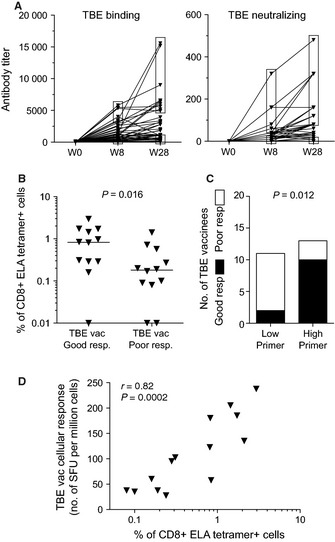
Assessment of *in vitro* CD8^+^ T‐cell priming capacity in elderly adults. (A) Binding and neutralizing antibody titers specific for TBEv in elderly (>70 years old) adults before and at weeks 8 and 28 after the first immunization. Top and bottom quartiles of titer values (indicated by the upper and lower frames respectively) at weeks 8 or 28 were used to define good (*n* = 12) and poor (*n* = 12) TBE vaccine responders, respectively. (B) Frequencies of ELA/HLA‐A2 tetramer^+^ CD8^+^ T‐cells after *in vitro* priming in good or poor TBE vaccine responders. Bars indicate median values. The statistical comparison was conducted using the Mann–Whitney *U*‐test. (C) Association between *in vitro* CD8^+^ T‐cell priming efficacy prior to TBE vaccination and TBE vaccine responsiveness based on anti‐TBEv antibody titers. Statistical significance was assessed using the chi‐square test. (D) Correlation between *in vitro* CD8^+^ T‐cell priming efficacy prior to TBE vaccination and the *de novo* TBE‐specific T‐cell responses determined by IFN‐γ ELISpot at week 26 postimmunization. The correlation was determined using Spearman's rank test.

Initially, we studied a group of HLA‐A2^+^ elderly individuals who mounted a primary immune response *in vivo,* upon vaccination for the first time against tick‐borne encephalitis virus (TBEv). The individuals selected for this study had never been exposed to TBEv as indicated by the absence of serum anti‐TBEv antibodies prior to vaccination. *De novo* humoral and cellular immune responses to TBE vaccination were monitored at weeks 8 and 28 or at week 26 postimmunization, respectively, and compared to baseline values. Among forty HLA‐A2^+^ vaccinees, we could define good (*n* = 12) and poor (*n* = 12) TBE vaccine responders, referring to donors with both TBEv binding and neutralizing antibody levels at weeks 8 or 28 postimmunization within the top and bottom quartiles of all titer values, respectively (Fig. 2A). Assessment of CD8^+^ T‐cell priming efficacy was performed in these individuals using our *in vitro* approach. Good TBE vaccine responders displayed significantly stronger CD8^+^ T‐cell priming efficacies *in vitro* compared to poor responders (Fig. 2B). Moreover, the frequency of ELA/HLA‐A2 tetramer^+^ cells after *in vitro* expansion assessed at day 0 (i.e., prevaccination) was associated with subsequent TBE vaccine responsiveness: high primers with ELA/HLA‐A2 tetramer^+^ cell expansions above the median frequency (i.e., 0.28% of tetramer^+^ cells within CD8^+^ T lymphocytes) at day 0 constituted a significantly greater proportion of good TBE vaccine responders compared to low primers (Fig. 2C). In addition, we found a direct correlation between *in vitro* CD8^+^ T‐cell priming capacity at day 0 and *ex vivo* TBE cellular responses measured at week 26 postimmunization in vaccinees who displayed a detectable TBE cellular response (*n* = 14) (Fig. 2D). Overall, although the mechanistic tie between responsiveness to a model antigen *in vitro* and to a vaccine *in vivo* is most likely indirect, these data indicate that the impairment of CD8^+^ T‐cell priming efficacy as measured *in vitro* in our assay reflects to some extent *in vivo* immune defects.

### Quantitative reduction of CD8^+^ T‐cell priming efficacy in the elderly

**Figure 3:**
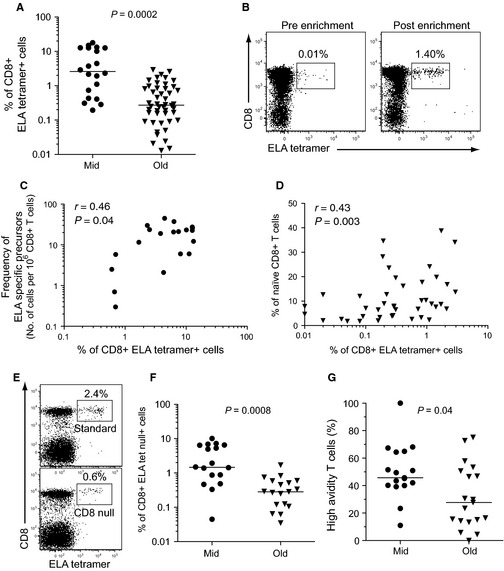
Assessment of qualitative attributes of CD8^+^ T‐cells primed *in vitro*. (A) Frequencies of ELA/HLA‐A2 tetramer^+^ CD8^+^ T‐cells after *in vitro* priming (day 10) in healthy, HLA‐A2^+^ middle‐aged (*n* = 20, Mid) and elderly (>70 years old) adults (*n* = 46, Old). (B) Representative flow cytometry plots showing ELA‐specific CD8^+^ T‐cell precursors in a healthy donor before and after enrichment from 10^8^ PBMCs. Percentages of ELA/HLA‐A2 tetramer^+^ cells within the CD8^+^ T‐cell population are indicated. (C) Correlation between ELA‐specific CD8^+^ T‐cell precursor (CD45RA^+^ CCR7^+^) frequency and ELA/HLA‐A2 tetramer^+^ CD8^+^ T‐cell frequency after *in vitro* priming in middle‐aged healthy adults (*n* = 20). (D) Correlation between naïve CD8^+^ T‐cell frequency and ELA/HLA‐A2 tetramer^+^ CD8^+^ T‐cell frequency after *in vitro* priming in healthy elderly (>70 years old) adults. Correlations were determined using Spearman's rank test. (E) Representative flow cytometry plots showing standard or CD8‐null tetramer staining to identify total or high‐avidity ELA‐specific CD8^+^ T‐cells, respectively. Percentages of ELA/HLA‐A2 tetramer^+^ cells within the CD8^+^ T‐cell population are indicated. (F) Frequencies of high‐avidity ELA‐specific CD8^+^ T‐cells in healthy middle‐aged (*n* = 17) and elderly (*n* = 19) adults with strong expansions (>0.4%) of total ELA/HLA‐A2 tetramer^+^ CD8^+^ T‐cells after *in vitro* priming. (G) CD8‐null/standard ratios for ELA/HLA‐A2 tetramer^+^ CD8^+^ T‐cells in healthy middle‐aged (*n* = 17) and elderly (*n* = 19) adults after *in vitro* priming. Bars indicate median values. Statistical analyses were conducted using the Mann–Whitney *U*‐test.

The magnitude of ELA/HLA‐A2 tetramer^+^ cells after *in vitro* expansion was used to compare antigen‐specific CD8^+^ T‐cell priming capacity in HLA‐A2^+^ healthy middle‐aged and elderly (>70 years old) adults. Using this approach, we found that the expansion of CD8^+^ T‐cells specific for our model antigen was significantly lower in elderly individuals compared to middle‐aged controls (Fig. 3A). This finding implies that advanced age is associated with quantitatively impaired CD8^+^ T‐cell priming. Recent studies in murine models suggest that the frequency of naïve T‐cell precursors correlates with the magnitude of the primary T‐cell response (Moon *et al*., 2007; Kotturi *et al*., 2008; Obar *et al*., 2008). Accordingly, we quantified naïve ELA‐specific CD8^+^ T‐cell precursor frequencies in a subset of healthy donors using an established procedure for the enrichment of CD45RA^+^ CCR7^+^ tetramer^+^ cells from PBMC samples via magnetic separation (Fig. 3B) (Alanio *et al*., 2010). A direct correlation was observed between the frequency of ELA/HLA‐A2 tetramer^+^ cells after *in vitro* expansion and the frequency of ELA‐specific CD8^+^ T‐cell precursors (Fig. 3C). Due to the high number of PBMCs required for antigen‐specific precursor quantification, the same approach was not possible in elderly individuals. Instead, we measured the frequency of total naïve (CD45RA^+^ CCR7^+^ CD27^+^) CD8^+^ T‐cells in these donors. A direct correlation was observed between the frequency of primed ELA/HLA‐A2 tetramer^+^ cells and the frequency of naïve CD8^+^ T‐cells in this group (Fig. 3D). Overall, these data support a relationship between the size of the naïve T‐cell pool and the efficacy of CD8^+^ T‐cell priming in humans. Accordingly, impaired CD8^+^ T‐cell priming in the elderly may be attributed, at least in part, to declining thymic output and a consequent reduction in naïve T‐cell frequencies.

In donors with large expansions of ELA‐specific CD8^+^ T‐cells after *in vitro* priming (i.e., > 0.4% ELA/HLA‐A2 tetramer^+^ CD8^+^ T‐cells), we also performed parallel experiments using CD8‐null ELA/HLA‐A2 tetramers (Fig. 3E). These reagents incorporate the compound D227K/T228A mutation into the α3 domain of the HLA‐A2 heavy chain, which abrogates CD8 coreceptor binding, thereby enabling the selective identification of high‐avidity antigen‐specific CD8^+^ T‐cells (Price *et al*., 2005; Wooldridge *et al*., 2009). High‐avidity CD8^+^ T‐cells are known to display superior functional (e.g., cytolytic and polyfunctional) attributes and greater efficacy against pathogens and tumors (Appay *et al*., 2008). In line with the results obtained using standard tetramers, significantly lower frequencies of primed CD8‐null ELA/HLA‐A2 tetramer^+^ CD8^+^ T‐cells were observed in elderly individuals compared to middle‐aged controls (Fig. 3F). Importantly, the CD8‐null/standard tetramer frequency ratios (i.e., the proportion of high‐avidity T‐cells among primed lymphocytes) were also lower in the elderly group (Fig. 3G). Thus, advanced age is associated with lower frequencies of avidity‐impaired primary responses, presumably as a result of repertoire perturbations within the naïve CD8^+^ T‐cell pool associated with a reduction in naïve T‐cell frequencies.

### Altered quality of *in vitro* primed CD8^+^ T‐cells originating from elderly individuals

**Figure 4:**
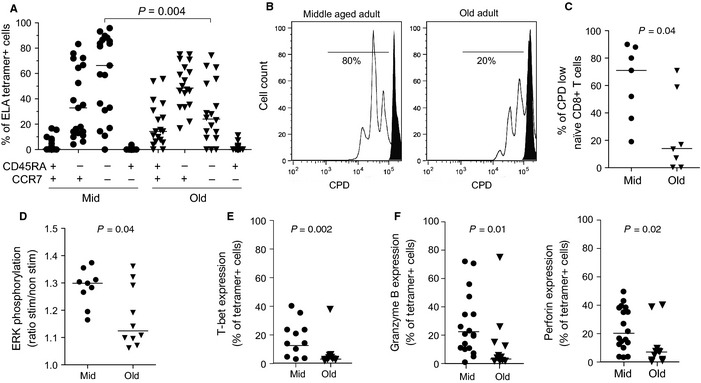
Quantitative and qualitative analysis of the naïve CD8^+^ T‐cell compartment. (A) Phenotypic distribution (based on CD45RA and CCR7 expression) of ELA/HLA‐A2 tetramer^+^ CD8^+^ T‐cells at day 10 postpriming in healthy middle‐aged and elderly adults. (B) Representative flow cytometry histograms showing CPD dilution in naïve (gated on CD45RA^+^ CCR7^+^ CD27^+^) CD8^+^ T‐cells from middle‐aged and elderly adults 4 days after stimulation with anti‐CD3/CD28 beads (in white) or medium alone (in black). (C) Percentages of CPD‐low naïve CD8^+^ T‐cells from middle‐aged and elderly adults 4 days after stimulation with anti‐CD3/CD28 beads. (D) Phosphorylation of ERK in naïve CD8^+^ T‐cells, from middle‐aged and elderly adults expressed as the MFI ratio under conditions of anti‐CD3/CD28 stimulation versus medium alone. (E) Intracellular T‐bet expression by ELA‐specific CD8^+^ T‐cells at day 10 postpriming from healthy middle‐aged adult and elderly PBMC samples. (F) Granzyme B and perforin expression by ELA‐specific CD8^+^ T‐cells at day 10 postpriming from healthy middle‐aged adult and elderly PBMC samples. Bars indicate median values. Statistical comparisons were conducted using the Mann–Whitney *U*‐test.

Next, we used our approach to assess qualitative features of antigen‐specific CD8^+^ T‐cells primed *in vitro,* comparing samples from healthy middle‐aged and elderly donors. Although the majority of ELA‐specific CD8^+^ T‐cells primed from middle‐aged adult samples displayed a CD45RA^−^ CCR7^−^ effector memory phenotype, expanded ELA/HLA‐A2 tetramer^+^ cells from elderly donor PBMCs were typically CD45RA^−^ CCR7^+^, a phenotype usually associated with less differentiated cells (Fig. 4A). This observation suggests less efficient activation of elderly naïve CD8^+^ T‐cells, and altered differentiation into effector memory cells, potentially due to qualitative cellular defects. Impaired CD8^+^ T‐cell priming may also be related to intrinsic defects in old naïve T‐cells and failure to expand in response to TCR‐mediated signals. We therefore tested the capacity of total naïve (CD45RA^+^ CCR7^+^ CD27^+^) CD8^+^ T‐cells from elderly or middle‐aged control PBMC samples to proliferate upon anti‐CD3/CD28 stimulation. Elderly naïve CD8^+^ T‐cells displayed a reduction in proliferative capacity, as illustrated by lower Cell Proliferation Dye (CPD) dilution 4 days after stimulation (Fig. 4B, C).

In addition, we assessed rapid phosphorylation of ERK upon anti‐CD3/CD28 stimulation. ERK is a central element of the TCR signaling complex, with phosphorylation resulting in the induction of AP‐1, an essential transcription factor required for optimal T‐cell activation and proliferation. Upon stimulation, ERK phosphorylation was weaker in elderly naïve CD8^+^ T‐cells compared to middle‐aged donor controls (Fig. 4D), suggesting impaired TCR signaling in these cells. We next analyzed the intracellular expression in primed CD8^+^ T‐cells of the T‐box transcription factor T‐bet (also known as Tbx‐21), which lies further downstream in the TCR signaling pathway and plays an important role in effector memory T‐cell differentiation (Glimcher *et al*., 2004). ELA‐specific CD8^+^ T‐cells expanded from elderly donor PBMCs exhibited significantly less T‐bet expression compared to CD8^+^ T‐cells primed from middle‐aged adult samples (Fig. 4E), further supporting the notion of altered TCR signaling in older individuals. In CD8^+^ T‐cells, T‐bet is a master regulator of the expression of effector molecules such as granzyme B and perforin. In line with their low T‐bet expression, CD8^+^ T‐cells primed from elderly donor PBMCs also displayed a significant reduction in granzyme B and perforin content compared to middle‐aged donor controls (Fig. 4F).

Overall, a quantitative shrinkage of the naïve T‐cell pool, together with qualitative defects in old naïve CD8^+^ T lymphocytes, exemplified by impaired TCR signaling and poor proliferative capacity, is likely to result in the generation of fewer and mostly paucifunctional effector CD8^+^ T‐cells. Together, these factors may contribute to a reduction in CD8^+^ T‐cell priming efficacy in the elderly.

## Discussion

Although studies performed in animal models point to a decline in the induction of *de novo* CD8^+^ T‐cell responses with old age, no corresponding human data are available. This is largely due to a lack of practical assays designed to assess priming capacity in humans. To overcome these limitations, we developed a simple and original *in vitro* approach to assess the efficacy of antigen‐specific T‐cell priming directly from human blood samples. We focused our study on naïve CD8^+^ T‐cell responses to the immunodominant melanoma antigen Melan‐A/MART‐1, which is restricted by the HLA‐A2 molecule. The highly immunogenic heteroclitic variant of Melan‐A/MART‐1, ELA, was used as a model antigen. Starting from donor PBMCs, the assay relates *in vitro* priming capacity to the induction of *de novo* immune responses *in vivo*; it thus represents a unique setting to compare T‐cell priming efficacy between HLA‐matched individuals. Using this approach, we found that elderly adults displayed a reduced ability to prime antigen‐specific CD8^+^ T‐cells. More importantly, however, the quality of these primed T‐cells was altered. On one hand, the induction of high‐avidity CD8^+^ T‐cells appeared to be particularly affected in the elderly. This is in line with a recent report describing higher proportions of low‐avidity virus‐specific memory CD8^+^ T‐cells in old individuals (Griffiths *et al*., 2013). On the other hand, primed CD8^+^ T‐cells derived from elderly donor samples displayed significantly lower levels of the cytotoxins perforin and granzyme B. Taking into account the importance of TCR avidity and cytolytic functions for CD8^+^ T‐cell efficacy (Appay *et al*., 2008), declining numbers of high‐quality T‐cells represent a particular drawback for cellular immune competence.

Multiple parameters determine the fate of T‐cells upon priming with antigen. Studies performed in mice have shown that the naïve T‐cell precursor frequency can greatly influence the induction of an effective T‐cell response (Moon *et al*., 2007; Kotturi *et al*., 2008; Obar *et al*., 2008). In line with these findings, we demonstrated that the *ex vivo* precursor frequency correlates with the magnitude of the *in vitro* primary T‐cell response. Furthermore, we observed a direct association between CD8^+^ T‐cell priming efficacy and the size of the naïve T‐cell pool in elderly individuals. This finding suggests that compromised CD8^+^ T‐cell priming efficacy in elderly individuals is related to a decrease in the size of the naïve CD8^+^ T‐cell compartment and associated repertoire perturbations. One study suggested that the diversity of the naïve CD4^+^ TCR repertoire is maintained throughout adulthood but then undergoes a precipitous decline beyond 70 years of age (Naylor *et al*., 2005). Due to the low frequency of naïve cells possessing high‐avidity TCRs (at least with respect to ELA specificity), the recruitment of effective clonotypes may be particularly affected by the restricted naïve T‐cell repertoire in old age. Thymic involution, dwindling thymic output and new naïve T‐cell production (Gruver *et al*., 2007), which all accompany aging, therefore likely play an important role in the loss of T‐cell priming efficacy observed with age.

Other causes of reduced CD8^+^ T‐cell priming efficacy also need to be considered, including intrinsic qualitative cellular defects, which may affect the activation and differentiation of primed CD8^+^ T‐cells in the elderly. For instance, our data indicate that old individuals exhibit impaired TCR signaling, associated with suboptimal proliferation of naïve CD8^+^ T‐cells. Potential defects in TCR signaling, as indicated by reduced ERK phosphorylation in naïve CD8^+^ T‐cells and lower T‐bet expression in primed cells, may account for less efficient activation of elderly naïve CD8^+^ T‐cells. Cellular processes such as differentiation into effector memory cells may consequently be altered, leading to the generation of T‐cells with limited functional properties, as we observed after *in vitro* priming. These qualitative cellular defects are reminiscent of observations from an aged mouse model of infection (Smithey *et al*., 2011). Our data are also consistent with recent findings by Li *et al*., who characterized an age‐related defect in TCR‐induced ERK phosphorylation and antigen sensitivity in old naïve human CD4^+^ T‐cells (Li *et al*., 2012). In this study, the defect in ERK phosphorylation was associated with reduced expression of miR‐181a in naïve CD4^+^ T‐cells with old age, resulting in increased DUSP6 activity and, ultimately, desensitization of the TCR cascade. It is unclear at present whether a similar mechanism is at work in old naïve CD8^+^ T‐cells. Furthermore, it remains to be determined whether the increased nonspecific homeostatic proliferation that characterizes naïve T‐cells in the elderly influences priming efficacy upon antigen encounter, potentially via effects on cellular metabolism and the acquisition of functional properties (Sauce *et al*., 2012).

The signals delivered to naïve T‐cells upon priming depend further on the nature of the antigen‐presenting cell. Increasing evidence suggests that the capacity of DCs to capture and process antigen is compromised with old age (Gupta, 2014). Other age‐related changes affecting DCs, for instance at the level of costimulatory protein expression and the speed of response to stress molecules (e.g., Toll‐like receptor ligands), were also highlighted in a recent report (Metcalf *et al*., 2014). In this study, the defective response to innate immune agonists (e.g., TLR4, TLR7/8, and RIG‐I ligands) resulted in reduced production of pro‐inflammatory cytokines and chemokines (e.g., TNF‐α, IL‐6, IL‐1β, IFN‐α, IFN‐γ, CCL2, and CCL7), contributing to impaired T‐cell proliferation. Given the importance of DCs in the process of T‐cell priming, these observations imply that perturbations of the DC compartment may also contribute to altered CD8^+^ T‐cell priming capacity. Reduced T‐cell priming efficacy with old age may therefore be related to the loss of primary immune resources, combining alterations in both naïve T‐cell and DC frequency and function. As our present approach does not allow us to fully discriminate between the different factors affecting T‐cell priming efficacy, further studies are needed to decipher the precise mechanisms involved. In addition, as the present study focused on the priming of CD8^+^ T‐cells specific for the Melan‐A/MART‐1‐ model antigen, it will be important to extend our observations to other specificities. Our *in vitro* approach offers the potential to study responses directed against other epitopes and different HLA restrictions. However, given the *in vitro* nature of these observations and the contingent necessity to interpret them with caution, the altered quality of *de novo* T‐cell responses in the elderly will need to be confirmed directly *in vivo*.

The decline of immune competence with advanced age emerges as a global phenomenon, with parallel alterations to the cellular, humoral, and innate arms of the immune system. In this context, the present work provides evidence of impaired *de novo* CD8^+^ T‐cell responses in elderly individuals. We propose that this phenomenon contributes to the loss of vaccine responsiveness and immune protection against pathogens and tumors in old humans.

## Experimental procedures

### Study subjects and samples

Two groups of HLA‐A2^+^ Caucasian volunteers were enrolled in this study: (i) middle‐aged (20 < age < 50) healthy adults (median age, 35 years); and (ii) elderly (>70 years old) healthy adults (median age, 78 years). Volunteers were screened for surface HLA‐A2 expression by flow cytometry using anti‐HLA‐A2 antibodies (BD Biosciences, San Diego, CA). Individuals undergoing immunosuppressive therapy were excluded from the study. Among the elderly, we studied an additional group of individuals who received a full TBE immunization course of three injections at weeks 0, 4, and 24 with a licensed inactivated whole virus vaccine (FSME Immun^®^; Baxter, Volketswil, Switzerland) as part of a clinical trial (NCT00461695). All individuals were healthy (no chronic diseases, ≤ one medication) and seronegative for TBEv. Assays of immune reactivity were conducted prior to vaccination, and at weeks 8 and 28 postvaccination for humoral and at week 26 for cellular responses. The study was approved by the Comité de Protection des Personnes of the Pitié Salpétrière Hospital (Paris) and by the Cantonal Ethics Committe (Zurich). All participants provided written informed consent. Venous blood samples were drawn into anticoagulant tubes, and PBMCs were isolated by density gradient centrifugation according to standard protocols.

### *In vitro* priming of antigen‐specific CD8^+^ T‐cell precursors

Naïve precursors specific for the HLA‐A2‐restricted epitope ELAGIGILTV (ELA) were primed *in vitro* using a previously published method with minor adaptations (Martinuzzi *et al*., 2011). Briefly, thawed PBMCs were resuspended in AIM medium (Invitrogen, Carlsbad, CA), plated out at 2.5 × 10^6^ cells/well in a 48‐well tissue culture plate, and stimulated with the peptide YTAAEELAGIGILTVILGVL, which contains the optimal epitope in heteroclitic form, at a concentration of 1 μm in the presence of FLT3 ligand (50 ng mL^−1^; R&D Systems, Minneapolis, MN). After 24 h (day 1), maturation was induced by the addition of a cytokine cocktail comprising TNF‐α (1000 U mL^−1^), IL‐1β (10 ng mL^−1^), IL‐7 (0.5 ng mL^−1^), and PGE2 (1 μm) (R&D Systems) (Martinuzzi *et al*., 2011). On day 2, fetal calf serum (FCS; Gibco, Carlsbad, CA) was added to reach 10% by volume per well. Fresh RPMI‐1640 (Gibco) enriched with 10% FCS was subsequently used to replace the medium every 3 days. The frequency and phenotype of ELA‐specific CD8^+^ T‐cells were typically determined on day 10. Purified naïve and memory CD8^+^ T‐cell subsets for priming experiments were obtained using T‐cell Enrichment Column Kits (R&D Systems).

### Flow cytometry reagents and procedures

Fluorochrome‐conjugated ELA/HLA‐A2 tetramers were produced and used as described previously (Price *et al*., 2005). The D227K/T228A compound mutation was introduced into the α3 domain of HLA‐A2 to generate CD8‐null tetramers, which enable the selective identification of high‐avidity antigen‐specific CD8^+^ T‐cells (Purbhoo *et al*., 2001; Wooldridge *et al*., 2009). The following directly conjugated monoclonal antibodies were used according to standard protocols: αCD8 APC‐Cy7 (Caltag, Carlsbad, CA); αCD27 AlexaFluor 700 (BioLegend San Diego, CA); αCD45RA ECD (Beckman Coulter, Miami, FL); α‐perforin‐BD48 FITC (Abcam, Cambridge, MA); α‐T‐bet AlexaFluor647 (eBiosciences, San Diego, CA); and αCCR7 PE‐Cy7, αHLADR PE‐CF594, αCD11c V450, αCD123 PE‐Cy7, α‐granzyme B V450, and lineage cocktail (including αCD3, αCD14, αCD16, αCD19, αCD20, αCD56) FITC (BD Biosciences). Samples were acquired using a Fortessa flow cytometer (BD Biosciences). Intracellular staining for T‐bet was performed using the Transcription Factor Buffer Set (BD Pharmingen, San Diego, CA) according to the manufacturer's instructions. Intracellular staining for granzyme B and perforin‐BD48 was compatible with this procedure. Data analysis was conducted with facsdiva 7.0 (BD Biosciences) and flowjo v9 (TreeStar Inc.) software. *Ex vivo* frequencies of ELA‐specific precursors were determined from pre‐enriched PBMC samples according to published procedures (Alanio *et al*., 2010; Iglesias *et al*., 2013).

### Analysis of TBEv‐specific humoral and cellular immune responses

TBEv‐specific antibody titers were measured before (week 0), during (week 8), and after (week 28) the TBEv vaccination course by ELISA and TBEv‐neutralization according to published protocols (Stiasny *et al*., 2012). The TBEv‐specific cellular immune response was assessed at weeks 0 and 26 by IFN‐γ enzyme‐linked immunosorbent spot (ELISpot) assay using pools of overlapping peptides for all structural proteins of TBEv. Briefly, 2 × 10^5^ thawed PBMCs/well from the same donor at week 0 and week 26 were stimulated in α‐IFN‐γ (clone 1‐D1K; Mabtech, Nacka Strand, Sweden)‐coated 96‐well ELISpot plates (MAIP S45; Millipore) for 18 h with 2 × 10^4^ freshly generated autologous monocyte‐derived DCs. For antigen‐specific stimulation, five pools of overlapping peptides encompassing all structural proteins of TBEv were used at a final concentration of 1μg mL^−1^ (15‐mer peptides overlapping by 5 amino acids; BMC Microcollections, Germany). Washed plates were then incubated with α‐IFN‐γ‐biotin (7‐B6‐1; Mabtech) followed by streptavidin–alkaline phosphatase (Mabtech), developed with color reagents (170‐6432; Bio‐Rad , Marnes‐la‐Coquette, France) and analyzed in an automated ELISpot reader (AID). The number of spot‐forming units (SFU) was calculated after subtraction of the unstimulated control.

### Proliferation assay and ERK phosphorylation

Total PBMCs were stained with Cell Proliferation Dye eFluor 450 (CPD; Affymetrix, eBiosciences) at 20 μm for 10 min, then washed and stimulated with Dynabeads^®^ Human T‐Activator CD3/CD28 (Life Technologies, Carlsbad, CA). Three days later, the percentage of CPD‐low cells was evaluated within naïve (CD45RA^+^ CCR7^+^ CD27^+^) CD8^+^ T‐cells by flow cytometry. To assess ERK1 and ERK2 phosphorylation, PBMCs were stained with T‐cell differentiation surface markers for 15 min at room temperature and then exposed to Dynabeads^®^ Human T‐Activator CD3/CD28 (Life Technologies) for 2 min at 37°C. Cells were subsequently washed and fixed in BD Cytofix Fixation Buffer (BD Biosciences) for 10 min at 37°C and then permeabilized with BD Phosflow^™^ Perm Buffer III (BD Biosciences) for 20 min at 4°C. After washing, cells were stained intracellularly for 1 h at room temperature using BD Phosflow^™^ anti‐human ERK1/2 conjugated to AlexaFluor647 (BD Biosciences) and analyzed by flow cytometry.

### Statistical analysis

Univariate statistical analyses were performed using graphpad prism software. Groups were compared using the nonparametric Mann–Whitney or chi‐square tests. Spearman's rank test was used to determine correlations. *P* values below 0.05 were considered significant.

## Authorship contributions

OB designed the study, performed experiments, and analyzed the data; AL designed the study, performed experiments, and analyzed the data; KW performed experiments and analyzed the data; GA performed experiments and analyzed the data; AvB performed experiments and analyzed the data; KR performed experiments; TM performed experiments; EM contributed vital analytical tools; LP performed experiments and analyzed the data; KS designed the study and provided crucial cohort samples; DAP contributed vital analytical tools and wrote the paper; RM designed the study and contributed vital analytical tools; DS designed the study and provided crucial cohort samples; UK designed the study and provided crucial cohort samples; and VA designed the study, analyzed the data, and wrote the paper.

## Disclosure of conflict of interest

TBE vaccine (FSME Immun^®^) was provided free of charge by the manufacturer (Baxter, Austria). AvB, KW, and UK have received travel grants and an unrestricted educational grant from Baxter (Austria). AL, RM, and VA are inventors of patent #EP14305080 entitled ‘Methods for testing T‐cell priming efficacy in a subject’ filed on January 21, 2014. All other authors declare that they have no competing financial interests.

## Funding

This work was supported by the French Agence Nationale de la Recherche (ANR; projects ANR‐09‐JCJC‐0114‐01 and ANR‐14‐CE14‐0030‐01), the Fondation Recherche Médicale (project DEQ20120323690), the Swiss National Science Foundation (grant PP0033‐110737 to UK), the AETAS Foundation (Geneva, Switzerland), the Promedica Foundation (Chur, Switzerland), IRGHET, and INSERM‐Transfert. DAP is a Wellcome Trust Senior Investigator, and RM is an INSERM Avenir and APHP‐INSERM Contrat Hospitalier de Recherche Translationelle Investigator.
